# Can Patient-Reported Outcomes Measurement Information System® (PROMIS) measures accurately enhance understanding of acceptable symptoms and functioning in primary care?

**DOI:** 10.1186/s41687-020-00206-9

**Published:** 2020-05-20

**Authors:** Ryan P. Jacobson, Daniel Kang, Jeff Houck

**Affiliations:** George Fox University, 414 N. Meridian St., Newberg, Oregon, USA

**Keywords:** Patient-reported outcomes, Primary care, PROMIS, PASS scores, Reference values

## Abstract

**Background:**

Value-based healthcare models will require prioritization of the patient’s voice in their own care toward better outcomes. The Patient-Reported Outcomes Measurement Information System® (PROMIS) gives patients a voice and leads providers to actionable treatments across a broad range of diagnoses. However, better interpretation of PROMIS measures is needed. The purpose of this study was to evaluate the accuracy of PROMIS Physical Function (PF), Self-Efficacy for Managing Symptoms (SE), Pain Interference (PI), Fatigue, and Depression measures to discriminate patient acceptable symptom state (PASS) in primary care, determining if that accuracy is stable over time and/or retained when PROMIS score thresholds are set at either ½ or 1 SD worse than the reference population mean.

**Methods:**

Primary care patients completed the five PROMIS measures and answered the PASS yes/no question at intake (*n* = 360), 3–14 days follow-up (*n* = 230), and 45–60 days follow-up (*n* = 227). Thresholds (optimal, ½ SD, and 1 SD worse than reference values) for PROMIS T-scores associated with PASS were determined through receiver-operator curve analysis. Accuracy was calculated at the three time points for each threshold value. Logistic regression analyses were used to determine combinations of PROMIS measures that best predicted PASS.

**Results:**

PROMIS PF, SE, PI, and Fatigue optimal score thresholds (maximizing sensitivity and specificity) yielded area under the curve values of 0.77–0.85, with accuracies ranging from 71.7% to 79.1%. Accuracy increased minimally (1.9% to 5.5%) from intake to follow-ups. Thresholds of 1 SD worse than the mean for PROMIS PF and PI measures and ½ SD worse for SE and Fatigue overall retained accuracy versus optimal (+ 1.3% to − 3.6%). Regression models retained SE, PI, and Fatigue as independent predictors of PASS, and minimally increased accuracy to 83.1?%.

**Conclusions:**

This study establishes actionable PROMIS score thresholds that are stable over time and anchored to patient self-reported health status, increasing interpretability of PF, SE, PI, and Fatigue scores. The findings support the use of these PROMIS measures in primary care toward improving provider-patient communication, prioritizing patient concerns, and optimizing clinical decision making.

## Background

Value-based healthcare models and many current health systems are moving toward routine collection of patient self-reported health data [1–3]. Patient-reported outcome measures (PROMs) represent an important component of outcomes assessment in value-based care toward comprehensive healthcare decision making [4]. Because PROMs give patients a voice in their own healthcare decision making [1, 5], such measures might improve provider-patient communication and clinical decision making [6]. In the primary care setting, many symptoms go under-recognized as pressing medical needs drive provider-patient discussion, and both parties neglect to bring up other potentially important health concerns [7]. Studies evaluating the impact of PROMs on care have shown increased patient-initiated discussion of symptoms that might have not otherwise been brought up [5, 8]. However, a key barrier to routine use of PROMs by providers is limited interpretability, with patient-reported data being viewed as not accurate or actionable [5, 8, 9]. Therefore, useful interpretation of scores is necessary for provider adoption of PROMs in clinical practice.

Actionable PROMs that are agnostic to disease, detect a range of severity, and have low patient burden may meaningfully assist primary care providers in managing chronic and acute symptoms. The Patient-Reported Outcomes Measurement Information System® (PROMIS) is a set of over 300 person-centered measures of symptoms and functioning in the domains of physical, mental, and social health [10]. An advantage over common legacy PROMs that capture responses regarding a specific condition is that PROMIS measures are applicable irrespective of diagnosis. Severity of symptoms or functioning is referenced to the US population or to patients with chronic conditions, detecting both worsening and improving status for individuals at all levels of health [11–14]. Administration of PROMIS measures via computer adaptive testing minimizes patient burden (average 44 to 65 s per measure), increasing feasibility of gauging patient health across multiple domains [11, 15, 16]. These advantages make PROMIS a good choice for the wide variety of patients and multi-system health complaints typical in primary care. Enhancing interpretability of select PROMIS measures in this setting will inform future clinical implementation of PROMIS toward improved provider-patient communication and clinical decisions [6, 9, 17, 18].

One way to improve interpretability of PROMIS scores is to threshold those scores against an anchoring question that captures a health construct valuable to patients. The *patient acceptable symptom state* (PASS) is a person-centered Yes/No question shown to have high discriminatory value as an anchoring question for scales of pain, disease activity, and functional level in many varied patient populations [19–22]. A PASS Yes response demarcates the level of symptoms and functioning beyond which a patient considers themselves well [17, 19, 23]. Using PASS, a threshold score value can be identified for any given PROMIS measure above which patients would likely report acceptable health status. One study in primary care musculoskeletal patients identified threshold scores for PROMIS Physical Function (PF), Self-Efficacy for Managing Symptoms (SE), and Pain Interference (PI) that discriminated PASS status with > 70% accuracy at the initial assessment [18]. Other studies in orthopedic services showed very high sensitivity or specificity discriminating PASS using PROMIS PF, PI, and Depression scores [17, 24]. PROMIS thresholds for acceptable/unacceptable symptoms and functioning would be useful to guide provider-patient discussion and determine patient priorities.

Previous attempts to incorporate PROMIS measures in physician services have had mixed results. Simple visual feedback of scores to primary care providers did not lead to improved patient interactions or outcomes [7]. However, the utility of the measures in identifying symptoms not easily detected in primary care (i.e. sleep, pain, anxiety, depression, and low energy/fatigue) was supported. Additionally, it was shown that many persistent symptoms co-occurred, suggesting that PROMs may save providers time trying to understand the multi-system symptoms and functional deficits patients are experiencing [7]. Training on PROMIS score interpretation in a rheumatology service resulted in discussion of scores with patients in 76% of visits, with very high provider confidence in the PROM data [6]. Such previous studies selected measures of symptoms that were common but often difficult to track in primary care [6, 7, 25]. Alternatively, a set of PROMIS measures might be selected as relevant to a broad range of primary care patient complaints (e.g. musculoskeletal, cardiac, metabolic/endocrine), including biomedical variables (physical function, pain, fatigue) and psychosocial variables (self-efficacy, depression). Previous studies have demonstrated utility of PROMIS measures directly in primary care [18] and for patients who frequent primary care [6, 17, 24]. To further improve primary care provider utility, understanding when PROMIS scores are likely unacceptable to patients would improve interpretation for clinical decisions.

Referenced to the US population or other patients with a variety of chronic conditions, PROMIS measures provide a novel window into severity of symptoms and functioning that may be useful for prioritizing patient complaints. Recent work in cancer has categorized severity for three PROMIS measures, with “mild” and “moderate” severity defined across score ranges of ½, 1, or 1½ standard deviation (SD) [9]. However, no study to date has sought to determine PROMIS score thresholds for acceptable/unacceptable symptoms and functioning in a primary care population, across multiple health domains, at multiple time points in care. Hence, the purposes of this study were to determine the following: 1) the extent to which PROMIS PF, SE, PI, Fatigue, and Depression measures are able to discriminate PASS status at intake, 3–14 days, and 45–60 days follow-up after a primary care encounter; 2) if the accuracy of PROMIS score thresholds to discriminate PASS status changes across 3–14 day and 45–60 day follow-ups; 3) what degree of accuracy is retained when PROMIS score thresholds are set at either ½ or 1 SD worse than the reference population mean; and 4) which combinations of PROMIS measures increase accuracy in discriminating PASS status when compared to an individual PROMIS measure.

## Methods

This was a longitudinal study of consecutive patients presenting to primary care in a rural, hospital based outpatient clinic between May 2018 and November 2018. Patients with all diagnoses and complaints signed informed consent to participate in the study in compliance with an IRB approved protocol (IRB #: STUDY2018000257), with all patient information anonymized. Data for PROMIS and PASS were collected in-person at intake, and then over the phone at 3–14 days and 45–60 days follow-ups. Inclusion criteria were minimal with all patients 18 years or older invited to participate. There were no other inclusion or exclusion criteria.

### Patient-reported outcome measures

Patients were administered PROMIS PF, SE, PI, Fatigue, and Depression measures and the PASS question, consistent with previous studies [18, 26]. PASS is a single-item question denoting the level of symptoms and functioning beyond which an individual considers their health status acceptable. Patients in this study responded Yes or No to the anchoring question, “Taking into account all the activities you have during your daily life, your level of pain, and also your functional impairment, do you consider that your current state is satisfactory?” [17, 19, 23, 27] The five PROMIS measures were administered via computer adaptive testing using the HealthMeasures iPad app (Glinberg & Associates, Inc). All PROMIS items administered in this study offer five polytomous response options, reflecting degrees of the trait being measured [15, 28, 29]. The PF v1.2 measure assesses functioning in mobility, use of arms and body, and capability in instrumental activities of daily living, with higher scores representing better functioning [30]. The SE v1.0 measure assesses confidence in controlling symptoms during work, play, sleep, and relationships, with higher scores representing better self-efficacy [31]. The PI v1.0 measure assesses the extent to which pain impacts daily life, with lower scores representing less pain interference [32]. The Fatigue v1.0 measure assesses experience and impact of fatigue on all daily activities, with lower scores representing less fatigue [33]. The Depression v1.0 measure assesses mood, view of self, social aspects, affect, and engagement, with lower scores representing less depression [34]. The PF v1.2, PI v1.0, Fatigue v1.0, and Depression v1.0 measures were each calibrated and validated on the general US population, with scores reported as a T-score with mean of 50, SD of 10 [30, 32–34]. The reference population for the SE v1.0 measure is patients with chronic conditions, again with a T-score mean of 50, SD of 10 [31].

The PROMIS measures and PASS question were administered at follow-up via phone by paid research assistants not otherwise involved in the study. Telephone administration of multiple PROMIS measures has been employed previously in a population-based study of 778 individuals with prostate cancer [35]. Callers received ongoing training to obtain accurate responses with the intent of minimizing caller influence on patient responses. Training included: 1) a standardized phone script, 2) initial practice on mock calls, 3) supervision of the initial 5–10 patient calls, and 4) intermittent feedback when the standardized script was difficult to apply. All calls were conducted directly with patients (no proxies).

### Chart review

Study personnel were trained in extracting information from the electronic medical record, including age, gender, height, weight, body mass index, primary diagnosis category, and other comorbidities (noted in patient problem lists). Comorbidities were categorized using the top 20 non-fatal chronic conditions [36] then collapsed into broad condition categories (e.g. metabolic/endocrine, cardiovascular, musculoskeletal, integumentary).

### Data analysis

Descriptive statistics from intake were used to describe sample characteristics, including diagnosis and PROM data. Inferential statistics were used for all other analyses with an alpha level set at *p* ≤ .05. Receiver operator characteristic (ROC) curves were used to determine area under the curve (AUC) for PROMIS measures’ ability to discriminate PASS status at intake, 3–14 days, and 45–60 days follow-ups. The 95% confidence interval for each AUC was calculated with acceptable AUC values defined to be 0.70–0.79 and “excellent” values at ≥0.80, as previously described [37]. To determine adequate sample size, the minimum acceptable AUC of 0.70 was used in the power analysis [38]. For a sample of 350, a proportion of 50% PASS Yes, yields a lower bound of the AUC confidence interval of 0.64. Therefore, this sample size would detect AUC for the ROC analysis as low as 0.64, likely lower than what would be considered clinically meaningful [37, 38]. Based on the ROC curve data, the Youden index was used to determine T-score thresholds with optimal sensitivity/specificity values (i.e. maximized sensitivity and specificity). The Youden index identifies the point on the ROC curve that is the greatest vertical distance from the chance line (i.e. the diagonal), maximizing sensitivity, specificity, and overall accuracy [39]. Based on these optimal thresholds, accuracy of the five PROMIS measures to each discriminate PASS status was calculated using 2 × 2 cross-tabs tables for intake, 3–14 day, and 45–60 day follow-ups. Next, using thresholds of ½ and 1 SD worse than the reference population mean (e.g. for PI using T-scores of 55 and 60), accuracy was again calculated using 2 × 2 cross-tabs tables for each of the five PROMIS measures. For all thresholds, sensitivity and specificity were also calculated. Finally, logistic regression using forward conditional criteria for all PROMIS measures was used to determine the best independent predictors of PASS. Variables with a *p* value less than 0.05 were retained in the model. To evaluate the influence of certain patient characteristics, age, gender, and BMI were added to the model to determine if these significantly influenced the selected PROMIS measures’ prediction of PASS. Interaction effects between the five PROMIS measures were also explored. Spearman correlations between PROMIS measures are also reported.

## Results

Across the three time points—intake (*n* = 360), 3–14 day follow-up (*n* = 230), and 45–60 day follow-up (*n* = 227)—there was no missing data within respondents. Phone call data collection response rates were 63.8% at the 3–14 day follow-up, and 63.1% at the 45–60 day follow-up. Table 1 describes sample characteristics and PROM data for the 360 primary care patients who completed all PROMs at intake. Age ranged from 20 to 97 years old with a mean age of 66.9 (17.0), 52.2% female, and a mean BMI of 31.1 (9.2). Metabolic/endocrine, circulatory, musculoskeletal, or integumentary conditions comprised 62.2% of primary diagnoses, with a mean number of comorbidities of 5.5 (2.7). A majority of patients reported PASS Yes status (57.8%). Across the five PROMIS measures, T-scores ranged from 3 SD worse to 2.5 SD better than the reference population mean (50). The percentage of patients with PROMIS T-scores at least ½ SD worse are reported for each measure, ranging from 34.4% to 58.6%, with 62.5% of patients being at least ½ SD worse on two or more measures.

**Table 1:** Sample characteristics and patient-reported outcomes data for all patients at intake (*n* = 360)

Characteristic / outcome variable	Value
Age, years	
mean (SD)	66.9 (17.0)
range	20–97
Female, *n* (%)	188 (52.2)
BMI	
mean (SD)	31.1 (9.2)
range	14.0–70.9
Primary diagnosis category, *n* (%)	
metabolic /endocrine	75 (20.8)
circulatory	67 (18.6)
musculoskeletal	63 (17.5)
integumentary	19 (5.3)
# Comorbidities	
mean (SD)	5.5 (2.7)
range	0–13
Patient Acceptable Symptom State Yes, *n* (%)	208 (57.8)
PROMIS measure T-scores	
Physical Function	
mean (SD)	43.0 (9.4)
range	20.0–73.3
½ SD worse^a^, *n* (%)	211 (58.6)
Self-Efficacy for Managing Symptoms	
mean (SD)	45.9 (7.8)
range	26.5–68.7
½ SD worse^a^, *n* (%)	175 (48.6)
Pain Interference	
mean (SD)	56.3 (8.7)
range	38.7–76.4
½ SD worse^a^, *n* (%)	204 (56.7)
Fatigue	
mean (SD)	54.4 (9.2)
range	24.3–76.0
½ SD worse^a^, *n* (%)	177 (49.2)
Depression	
mean (SD)	51.5 (9.1)
range	34.2–78.1
½ SD worse^a^, *n* (%)	124 (34.4)
Multiple PROMIS measures ½ SD worse^b^, *n* (%)	
2 measures	44 (12.2)
3 measures	50 (13.9)
4 measures	55 (15.3)
all 5 measures	76 (21.1)

*Abbreviations: SD* Standard deviation, *BMI* Body mass index, *PROMIS* Patient-Reported Outcomes Measurement Information System

^a^Number of patients with T-scores at least ½ SD below mean 50 on that measure

^b^Number of patients at least ½ SD below mean 50 on multiple measures

**Fig. 1:**
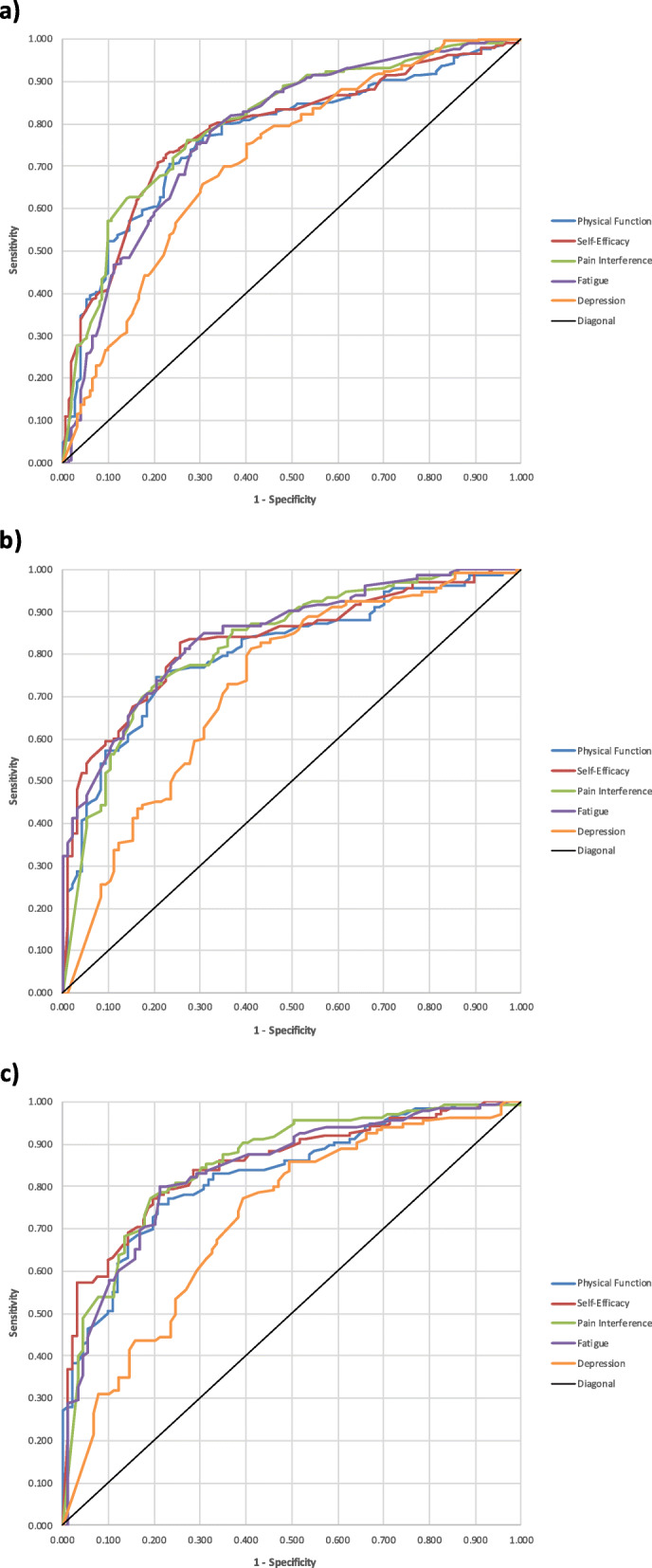
Receiver-operator characteristic curves for the five PROMIS measures’ ability to discriminate patient acceptable symptom state (PASS): **(a)** intake, **(b)** 3–14 day follow-up, **(c)** 45–60 day follow-up

The ROC curve (Fig. 1a–c) analysis revealed that all five PROMIS measures discriminated PASS status with significant AUC values at the *p* < .001 level (Table 2). For PROMIS PF, SE, PI, and Fatigue measures, AUC values were 0.77 to 0.81 at intake and 0.81 to 0.85 across the two follow-ups. For Depression, AUC values remained lower at 0.72 across all three time points, with 95% confidence intervals dropping below 0.70.

**Table 2:** Receiver operator curve analysis for intake (*n* = 360), 3–14 day follow-up (*n* = 230), and 45–60 day follow-up (*n* = 227)

PROMIS Measure	AUC (95% CI)^a^
Physical Function	
intake	0.77 (0.72–0.82)
3–14 day	0.81 (0.75–0.86)
45–60 day	0.83 (0.77–0.88)
Self-Efficacy for Managing Symptoms	
intake	0.78 (0.74–0.83)
3–14 day	0.83 (0.78–0.88)
45–60 day	0.85 (0.80–0.90)
Pain Interference	
intake	0.81 (0.76–0.85)
3–14 day	0.82 (0.77–0.88)
45–60 day	0.85 (0.80–0.90)
Fatigue	
intake	0.78 (0.73–0.83)
3–14 day	0.85 (0.80–0.89)
45–60 day	0.84 (0.78–0.89)
Depression	
intake	0.72 (0.66–0.77)
3–14 day	0.72 (0.66–0.79)
45–60 day	0.72 (0.65–0.79)

*Abbreviations: PROMIS* Patient-Reported Outcomes Measurement

Information System, *AUC* Area under the curve, *CI* Confidence interval

^a^All AUC values *p* < .001

Based on optimal T-score thresholds, accuracy discriminating PASS status increased between intake and follow-up for all five PROMIS measures (Table 3). The increase for PF was 4.5–4.8%, SE 3.9–5.5%, PI 1.9–2.4%, Fatigue 4.3–4.8%, and Depression 1.9–3.5%. For PF, SE, PI, and Fatigue, optimal thresholds yielded accuracy values of 71.7–73.6% at intake and 75.5–79.1% across follow-ups, with sensitivity and specificity ≥0.71 at intake (up to 0.79) and ≥ 0.73 across follow-ups (up to 0.83). For Depression, optimal threshold accuracy was 68.3% at intake, and ≤ 71.8% across follow-ups, with sensitivity and/or specificity dropping below 0.70 for all three time points.

**Table 3:** T-score thresholds^a^ accuracy at intake (*n* = 360), 3–14 day follow-up (*n* = 230), and 45–60 day follow-up (*n* = 227)

PROMIS Measure	Optimal			½ SD worse		1 SD worse	
	*Threshold*	*Accuracy*,%	*Sn, Sp*	*Accuracy*,%	*Sn, Sp*	*Accuracy*,%	*Sn, Sp*
Physical Function							
intake	42.9	71.7	0.71, 0.77	67.2	0.59, 0.83	***73.0***	0.80, 0.65
3–14 day	41.6	76.5	0.74, 0.79	70.4	0.59, 0.87	***73.9***	0.78, 0.68
45–60 day	41.4	76.2	0.76, 0.79	70.9	0.61, 0.88	***75.4***	0.78, 0.74
Self-Efficacy for Managing Symptoms							
intake	45.4	73.6	0.71, 0.79	***73.4***	0.72, 0.77	65.5	0.88, 0.35
3–14 day	46.1	79.1	0.83, 0.74	***75.7***	0.84, 0.64	65.7	0.96, 0.24
45–60 day	47.3	77.5	0.77, 0.80	***75.8***	0.84, 0.66	67.4	0.94, 0.30
Pain Interference							
intake	57.8	73.6	0.76, 0.73	70.3	0.63, 0.84	***72.2***	0.81, 0.61
3–14 day	55.9	76.0	0.74, 0.78	75.7	0.73, 0.79	***75.7***	0.86, 0.62
45–60 day	56.1	75.5	0.77, 0.81	74.3	0.71, 0.82	***76.5***	0.88, 0.62
Fatigue							
intake	57.1	73.6	0.78, 0.68	***69.7***	0.68, 0.73	72.5	0.88, 0.52
3–14 day	54.6	77.9	0.81, 0.73	***77.4***	0.81, 0.72	71.7	0.92, 0.44
45–60 day	54.9	78.4	0.80, 0.79	***78.4***	0.80, 0.79	73.6	0.93, 0.47
Depression							
intake	53.5	68.3	0.75, 0.60	***68.6***	0.80, 0.54	65.5	0.92, 0.29
3–14 day	50.7	71.8	0.81, 0.59	***70.0***	0.91, 0.41	63.1	0.95, 0.20
45–60 day	51.3	70.2	0.77, 0.61	***69.4***	0.90, 0.39	67.1	0.95, 0.26

*Abbreviations: PROMIS* Patient-Reported Outcomes Measurement Information System, *SD* Standard deviation, *Sn* Sensitivity, *Sp* Specificity

^a^Optimal T-score thresholds listed; better overall accuracy between ½ SD worse and 1 SD worse thresholds ***in italics*** for each measure

**Fig. 2:**
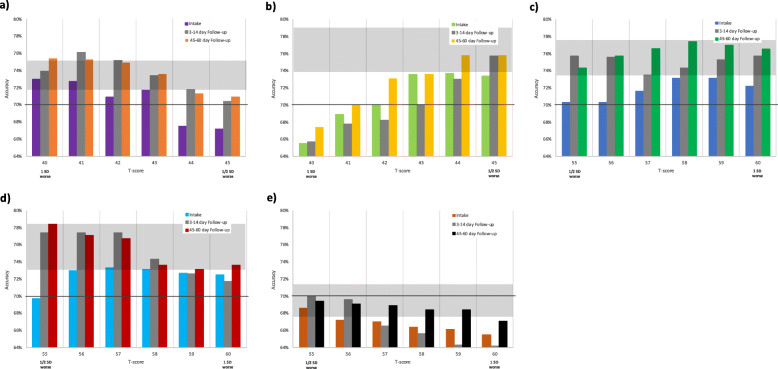
Accuracies for the five PROMIS measures’ ability to discriminate patient acceptable symptom state (PASS) across the three time points for all whole-number T-score thresholds from ½ to 1 standard deviation worse than the reference population mean score of 50: **(a)** Physical Function, **(b)** Self-Efficacy for Managing Symptoms, **(c)** Pain Interference, **(d)** Fatigue, **(e)** Depression. Gray strip indicates range of “optimal” T-score threshold accuracies for that PROMIS measure. Black horizontal line at minimum acceptable accuracy level

Based on T-score thresholds of either ½ or 1 SD worse than reference population mean, accuracy discriminating PASS status decreased no more than 3.6% versus optimal for all five PROMIS measures across all three time points (Table 3). For PF, choosing a threshold 1 SD worse (T-score 40) yielded accuracies of 73.0–75.4% (+ 1.3% to − 2.6% versus optimal) (Fig. 2a). For SE, ½ SD worse (45) yielded 73.4–75.8% (− 0.2% to − 3.4%), noting that the reference population is patients with chronic conditions (Fig. 2b). For PI, 1 SD worse (60) yielded 72.2–76.5% (+ 1.0% to − 1.4%), though ½ SD worse (55) yielded similar accuracies at 70.3–75.7% (− 0.3% to − 3.3%) (Fig. 2c). For Fatigue, intake accuracy was better retained (versus optimal) at 1 SD worse (60), yielding 72.5% (− 1.1%). However, Fatigue follow-up accuracies were better retained at ½ SD worse (55) at 3–14 day and 45–60 day follow-ups, yielding 77.4% (− 0.5%) and 78.4% (±0.0%), respectively (Fig. 2d). For Depression, ½ SD worse (55) yielded 68.6–70.0% (+ 0.3% to − 1.8%) (Fig. 2e).

Compared to individual PROMIS measures, regression analysis revealed minimal increases in accuracy discriminating PASS status using combinations of measures, and only at intake and 45–60 day follow-up only. PROMIS SE, PI, and Fatigue were retained in the final model for all time points. At intake the increase in accuracy was to 75.8% (+ 2.2% versus best accuracy of an individual PROMIS measure). At 3–14 day follow-up the accuracy was 79.1% (equivalent to the best accuracy of an individual PROMIS measure). At 45–60 day follow-up accuracy was 80.3% (again + 1.9% versus best accuracy of an individual PROMIS measure). Covariates of age, gender, BMI, and comorbidities did not increase accuracy of the model at intake or 3–14 day follow-up, though age did increase accuracy at 45–60 day follow-up to 82.8%. Looking for interaction effects, only a model using PF*SE [β = .002, exp.(β) = 1.002, *p* < .001] with PI [β = −.079, exp.(β) = .924, *p* = .001] increased accuracy at 45–60 day follow-up to 83.5%. Meanwhile, all correlations between the five PROMIS measures were statistically significant. Correlations between the PF, SE, PI, and Fatigue scores ranged from r = .61 to .64 at intake and r = .66 to .72 across the follow-up time points, with follow-up correlations between PF and SE (r = .70 to .72) and PF and PI (r = .69 to .71) being the highest. Depression showed overall lower values across all time points, correlating best with Fatigue (r = .52 to .62), less with SE and PI (r = .46 to .57), and least with PF (r = .36 to .44).

## Discussion

The identified T-score thresholds may assist primary care providers to prioritize which symptoms and functional areas are likely relevant to patients. Multiple studies across musculoskeletal conditions have shown that achieving an acceptable level of symptoms and functioning (i.e. PASS Yes) is of high value to patients [20–22, 40, 41]. This study shows that T-score thresholds of 1 SD worse than 50 for PROMIS PF and PI measures and ½ SD worse for SE and Fatigue measures are consistent with acceptable/unacceptable PASS status. In addition, the accuracy of these PROMIS measures in discriminating PASS status was relatively stable over the three time points assessed, suggesting that providers can make ongoing clinical decisions across follow-up visits based on these reported thresholds. Interestingly, the combination of PROMIS measures only marginally improved the discrimination of PASS status, suggesting clinically that the PF, SE, PI, and Fatigue measures used may each reflect patient experience independently. It is common for pertinent patient concerns to go undiscussed in primary care appointments [7]. Establishing PASS thresholds, stability over time, and only marginal gains in combining PROMIS measures informs the use of the selected PROMIS measures to facilitate provider-patient communication and elucidate otherwise undiscussed patient health concerns, improving clinical decision making.

This study sample comprised 360 primary care patients seeking care in a rural hospital-based clinic who consented to participate during the study period. Patients were 20–97 years of age with a mean BMI of 31.1 (9.2) and 5.5 (2.7) comorbidities, and with PROMIS T-scores ranging as low as 3 SDs worse than the reference population mean (Table 1). Administration of PROMIS measures and the PASS question occurred at various stages of ongoing primary care management for these patients, with all receiving usual care as prescribed by the provider. Therefore the sample outcomes likely best apply to a similar patient mix of primary care patients.

Four PROMIS measures—PF, SE, PI, and Fatigue—had AUC values of 0.77 to 0.85 for discriminating PASS status, indicating that perceived symptoms and functioning in these domains had relatively strong associations with self-appraised health status. The current sample was significantly more diverse than previous studies that focused on orthopedic problems [17, 18, 24]. Previous studies in orthopedic populations found similar AUC values (0.7–0.8) at initial evaluation with an orthopedic foot and ankle surgeon [24], in post-operative patients [17], and for a primary care musculoskeletal service [18]. Consistent with another study, the PROMIS Depression measure showed less ability to discriminate PASS (AUC < 0.72) [24]. The similarity of AUC values across studies suggest that the ability of PROMIS measures to discriminate acceptable and unacceptable health status with no additional health information is similar across different patient groups. This outcome increases confidence for providers in generalizing the identified PROMIS thresholds across patients.

For all five PROMIS measures, accuracy discriminating PASS status increased only 1.9%–5.5% at follow up (3–14 days and 45–60 days), thus exhibiting overall stability over time. The small increase in accuracy might reflect more thought put toward the measures by patients following initial exposure, and/or increased pertinence of factors as patients take action to address symptoms and functioning post-visit. Assuming some changes in disease status occurred over the follow up intervals, the stability supports the utility of thresholds for making ongoing clinical decisions across follow-up visits.

As anticipated based on a previous study [17], PROMIS PF, SE, PI, and Fatigue thresholds retained acceptable accuracy (≥72.2%) when applying T-score thresholds at ½ or 1 SD worse, comparable to optimal thresholds. Thus application of ½ or 1 SD thresholds clinically increases ease of use. Providers might even decide to choose between ½ and 1 SD thresholds based on what level of symptoms or functional deficit they wish to address, impacting how many patients receive follow up. For example, choosing to apply 1 SD worse thresholds for all four PROMIS measures would identify fewer patients but with greater certainty of true PASS No status (sensitivity 0.11–0.21 higher than ½ SD thresholds; Table 3).

Providers might also choose to apply the measure-specific PROMIS thresholds. Specific to PF and PI, using a T-score threshold 1 SD worse than the US mean for discriminating a patient’s likely self-reported PASS status (i.e. below 40 for PF, above 60 for PI) adequately retained accuracy across all three time points (Fig. 2 a and c). For PF, the 1 SD threshold was clearly better than ½ SD in discriminating PASS, based on both accuracy and sensitivity/specificity values (Table 3). This suggests that a threshold of 40 is a good threshold clinically for determining unacceptable patient-perceived function, achieving 73.0–75.4% accuracy. For PI, a ½ SD thresholds remained acceptably within 3.3% of optimal. However, the 1 SD worse threshold had higher sensitivity values (ranging from 81.4–88.1; Table 3), such that a higher percentage of patients who report a PI T-score of > 60 viewed their current health status as unacceptable. This suggests that PI T-scores > 60 might best coincide with patient priorities in discussing significant symptoms. For SE and Fatigue, applying a T-score threshold ½ SD worse (i.e. below 45 for SE, above 55 for Fatigue) retained overall accuracy best (Fig. 2 b and d), but again providers might instead choose 1 SD to focus on likely higher severity levels.

Advocates for targeting care argue that unacceptable symptoms or functioning, once identified, are candidates for specific interventions aimed at remediating these [42]. To make this practical, it’s likely that allied health practitioners that support primary care (e.g. pharmacists, behaviorists, and physical therapists) may need to develop cost effective care plans to compliment the primary care provider. In fact, current interdisciplinary models of care (e.g. medical home) [43] call for reorganization of primary care [3], and case studies of interdisciplinary primary care teams [26] demonstrate the feasibility of this approach.

The identified thresholds augment interpretation of guidelines from the HealthMeasures PROMIS website [44]. These general guidelines categorize severity for all measures in this study except SE, with T-scores ½-1 SD worse than 50 being “mild” and 1–2 SD worse than 50 “moderate.” Severity for SE is categorized such that T-scores 1 SD higher to 1 SD lower than 50 are “average” amongst patients with chronic health conditions, with 1–2 SD lower than 50 being “low.” The PASS thresholds identified in this study augment current interpretation of these measures and in some cases re-interpret the severity categorization. For example, this study suggests a SE T-score below 45 is likely unacceptable, versus average, amongst primary care patients with an average 5.5. (2.7) chronic comorbidities. Affirming the current categories, the PASS thresholds for PF, PI, and Fatigue essentially align with mild or moderate severity, depending on the measure. Multiple studies have used patient-informed benchmarking techniques for PROMIS [9, 45, 46], and discrepancies between patient and provider definitions of severity have been reported [9]. This study reinforces the need to establish PROMIS T-score thresholds in alignment with patient health experiences, toward improved interpretability for providers. During post-data review with providers in this study, it was noted that the interpretation of PROMIS scores at times changed care decisions. It was also noted that the PROMIS measures were more helpful with new patients, when providers were taking a subjective history for the first time. However, while the providers valued knowing when symptoms or functional deficits were moderate or severe, not having available direct treatments tied to addressing these deficits dampened their enthusiasm for the measures.

Combining the PROMIS outcome measures with or without select routine clinical variables only marginally improved the discrimination of PASS status. Although the regression analysis determined that PI, SE, and Fatigue were independent predictors of PASS status, accuracy was only marginally improved versus the most accurate single measure T-score thresholds. The PF measure may not have contributed to PASS status in the final model due to the higher correlations found with SE and PI (r = .69–.72), noting that PF has been shown to correlate highly with PI (r = .66 to .76) in orthopedic populations [24, 47]. However, PF and SE showed the only significant interaction, though again only resulting in a small increase in accuracy. This minimal effect on accuracy of determining patients with acceptable symptoms and functioning using multiple variables likely reflects the relatively equal importance patients attribute to each of the PROMIS PI, SE and Fatigue measures alone. This is an important finding given that a majority of patients are experiencing multiple symptoms and low functioning (Table 1). Therefore, assisting patients to achieve an acceptable health status likely involves achieving acceptable scores on each measure. Future studies may consider other analyses examining the influence of multiple unacceptable PROMIS measures to elucidate how more complex sets of symptoms and low functioning influence acceptable or unacceptable health status. Also, studies may examine socioeconomic status and other determinants of health which have demonstrated a significant influence on acceptable health status [24], in addition to how prognostic markers of disease severity may improve interpretation of PROMIS measures.

### Limitations

First, while this data comes from a consecutive sampling of primary care patients, it is still one of convenience from one rural, hospital-based outpatient service. Second, the sample was comprised of patients who were at various stages of care and who did not necessarily receive a controlled or specific intervention between intake and follow-up. Therefore, changes in disease status were not tracked and likely varied based on many factors. Third, while accuracies for PROMIS thresholds here are overall strong (> 70%), there were many patients who reported PROMIS T-scores below threshold and yet PASS Yes status. Other studies have demonstrated such reporting discrepancies in patients of lower income status or with diagnosed depression [24, 27], as well as in those with rheumatoid arthritis of longer duration [22, 48]. Hence, T-score thresholds reported here should be applied alongside other clinical findings. Finally, since the 3–14 and 45–60 day follow up included ~ 63% of respondents, it cannot be excluded that the small differences from baseline are due to attrition.

## Conclusions

This study improves interpretability of selected PROMIS measures by identifying specific thresholds (½ or 1 SD worse than the reference population mean) on acceptable health status, demonstrating that these thresholds are stable across time and showing each individual PROMIS measures to be useful for interpreting patient health status. Thresholds for acceptable symptoms tended to coincide with the mild to moderate symptom severity range for some measures (PF, PI, Fatigue). However, this data suggests the SE measure should be reinterpreted where T-scores below 45 are likely considered unacceptable rather than average. Of the measures selected, accuracy for PASS thresholds were similar except for Depression measure which was lower. The various analyses support the application of these PROMIS measure thresholds in primary care for optimizing provider-patient communication and clinical decision making.

## Data Availability

The datasets used and analyzed during the current study are available from the corresponding author on reasonable request.

## Abbreviations

AUC: Area under the curve
PASS: Patient acceptable symptom state
PF: Physical Function
PI: Pain Interference
PROMIS: Patient-Reported Outcomes Measurement Information System®
PROM: Patient-reported outcome measure
ROC: Receiver operator characteristic
SD: Standard deviation
SE: Self-Efficacy for Managing Symptoms
